# Epigenetic regulation of AURKA by miR-4715-3p in upper gastrointestinal cancers

**DOI:** 10.1038/s41598-019-53174-6

**Published:** 2019-11-18

**Authors:** Ahmed Gomaa, Dunfa Peng, Zheng Chen, Mohammed Soutto, Khaled Abouelezz, Alejandro Corvalan, Wael El-Rifai

**Affiliations:** 1 Department of Surgery, University of Miami Miller School of Medicine, Miami, Florida USA; 2 Department of Veterans Affairs, Miami Healthcare System, Miami, Florida USA; 3 Department of Medicine, University of Miami Miller School of Medicine, Miami, Florida USA; 4 Advanced Center for Chronic Disease, School of Medicine, Pontificia Universidad Catolica de Chile, Santiago, Chile

**Keywords:** Cancer, Gastric cancer

## Abstract

Aurora kinase A (AURKA) is frequently overexpressed in several cancers. miRNA sequencing and bioinformatics analysis indicated significant downregulation of miR-4715-3p. We found that miR-4715-3p has putative binding sites on the 3UTR region of AURKA. Upper gastrointestinal adenocarcinoma (UGC) tissue samples and cell models demonstrated significant overexpression of AURKA with downregulation of miR-4715-3p. Luciferase reporter assays confirmed binding of miR-4715-3p on the 3UTR region of AURKA. miR-4715-3p mediated a reduction in AURKA levels leading to G2/M delay, chromosomal polyploidy, and cell death. We also detected a remarkable decrease in GPX4, an inhibitor of ferroptosis, with an increase in cleaved PARP and caspase-3. Inhibition of AURKA using siRNA produced similar results, suggesting a possible link between AURKA and GPX4. Analysis of UGC samples and cell models demonstrated increased methylation levels of several CpG nucleotides upstream of miR-4715-3p. 5-Aza-2′-deoxycytidine induced demethylation of several CpG nucleotides, restoring miR-4715-3p expression, leading to downregulation of AURKA. In conclusion, our data identified a novel epigenetic mechanism mediating silencing of miR-4715-3p and induction of AURKA in UGCs. Inhibition of AURKA or reconstitution of miR-4715-3p inhibited GPX4 and induced cell death, suggesting a link between AURKA and ferroptosis.

## Introduction

Gastrointestinal cancers are the most common cause of cancer related deaths in the world^1^.

Upper gastrointestinal adenocarcinomas (UGCs, cancers of the stomach and esophagus) are poorly responsive to therapy with an unfavorable outcome. In 2018, approximately 40,000 new UGC cases were diagnosed in the United States with more than 20,000 deaths^2^.

microRNAs (miRNAs) are small noncoding RNAs that regulate gene expression through inactivation of mRNA^3^. Several molecular mechanisms regulate miRNA levels, including promoter methylation of the host gene or miRNA genes and post-transcriptional mechanisms^4^. Dysregulation of miRNAs’ expression mediates changes of key biological processes in cancer cells such as invasion, proliferation, and apoptosis^5^. Hypermethylation of promoter regions of anti-tumorigenic miRNAs mediates their downregulation, inducing expression of several oncogenes with activation of oncogenic signaling pathways in cancer cells^6–8^.

Several genetic and epigenetic mechanisms are involved in the pathogenesis of UGCs^9–12^. AURKA is a serine/threonine kinase that regulates the function of the mitotic spindle in normal cells^13–15^. AURKA overexpression is frequently upregulated in several cancer types such as breast, ovarian, liver, colorectal, gastric and esophageal tumors^9,16–19^. High levels of AURKA promote activation of several oncogenic factors, including c-MYC, NF-kB, and β-catenin with suppression of tumor suppressors, such as p53 and p73^20,21^. While genetic amplification of AURKA has been described in cancer, its occurrence is low and does not match the observed high frequency of AURKA overexpression in many cancer types. Epigenetic regulation of AURKA is poorly understood. Our miRNA-seq using human and mouse tissue samples pointed out several conserved miRNAs in mouse and human gastric cancers^22^. In this study, we investigated the role of miR-4715-3p in regulating AURKA levels in UGCs.

## Results

### miR-4715-3p is downregulated in upper gastrointestinal cancers (UGCs)

**Figure 1:**
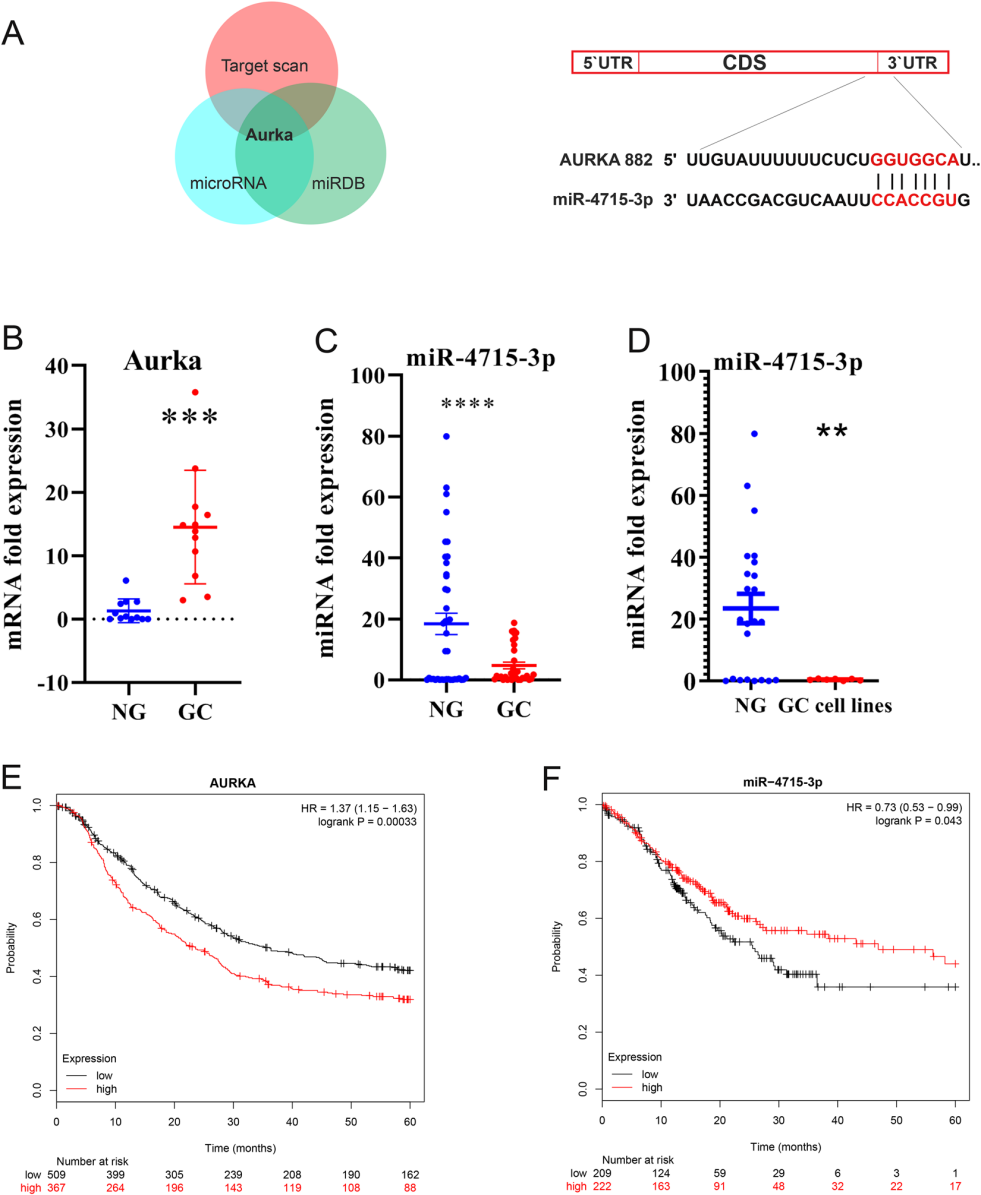
miR-4715-3p is downregulated in gastric cancer. (**A**) AURKA is a predicted target gene of miR-4715-3p in 3 different databases (www.TargetScan.org, www.microRNA.org, and www.miRDB.org) (left panel); a schematic drawing shows the specific binding sites of miR-4715-3p on AURKA 3′UTR (right panel). (**B**) AURKA mRNA is overexpressed in gastric cancer tissue samples. (**C**) qRT-PCR data showing miR-4715-3p expression levels in human gastric cancer (GC, n = 33) and non-tumor normal gastric (NG, n = 49) tissue samples. The horizontal bar indicates the median, Mann–Whitney test. (**D**) qRT-PCR data of miR-4715-3p expression in 15 human normal gastric samples and 6 human gastric cancer cell lines. (**E**) Kaplan–Meier analysis of AURKA-related overall survival in 876 human gastric cancer patients. ***P < 0.001. (**F**) Kaplan–Meier analysis of miR-4715-3p-related overall survival in 431 human gastric cancer patients. *P < 0.043.

We performed miRNA-seq using human and mouse tissue samples and identified a conserved miRNA signature in mouse and human gastric cancers^22^. We searched for miRNAs with predicted binding sites on AURKA 3′UTR using three online databases (www.TargetScan.org, www.microRNA.org, and www.miRDB.org). Among the downregulated microRNAs in gastric cancer, we found miR-4715-3p had a predictable target score of 86% for AURKA 3′UTR (Fig. 1A). We investigated the level of AURKA mRNA and miR-4715-3p expression in de-identified normal and tumor tissues. As shown in Fig. 1B, AURKA mRNA was significantly elevated in gastric tumor tissue samples, as compared with normal tissues (P < 0.001). Interestingly, miR-4715-3p was significantly downregulated in gastric tumor tissue samples and UGC cell lines, as compared with normal tissues (P < 0.001) (Fig. 1C,D). We detected a significant inverse correlation between up-regulated AUKRA and downregulated miR4715-3p in human gastric cancer tissue samples (Supplementary Fig. 1). Next, we analyzed the association between AURKA and survival in 806 gastric cancer patients using Kaplan–Meier survival plots. We found that patients with high expression levels of AURKA had significantly poorer survival than those with low levels (P < 0.001) (Fig. 1E). On the other hand, patients with low expression of miR-4715-3p had a significantly poorer prognosis than patients with high expression, using survival data of 431 gastric cancer patients (P < 0.043) (Fig. 1F).

### miR-4715-3p reconstitution induced downregulation of AURKA by directly targeting its 3′UTR

**Figure 2:**
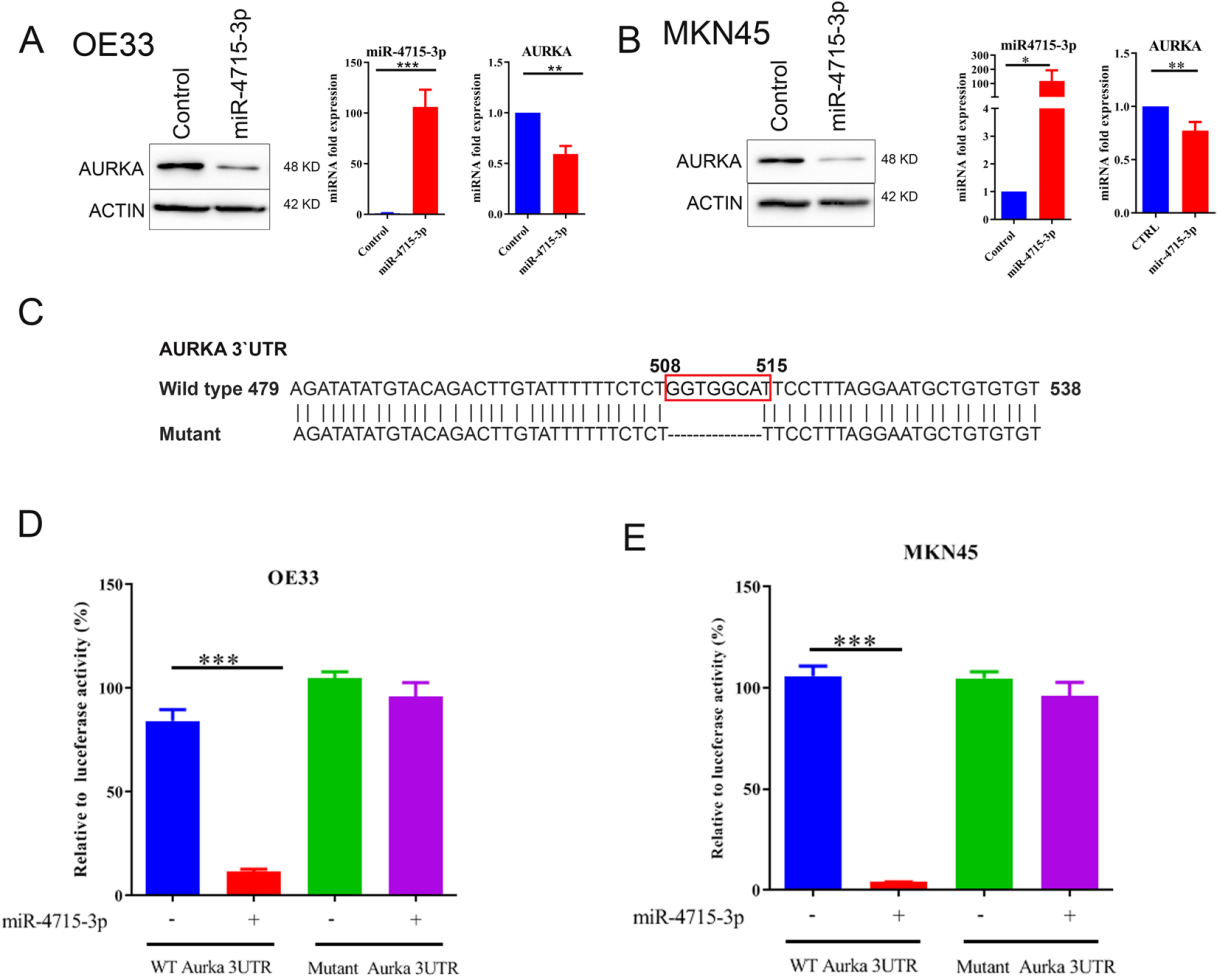
miR-4715-3p downregulates AURKA expression by directly targeting its 3′UTR in UGC cells. (**A**,**B**) AURKA and β-actin protein expression levels in control or after transient transfection of miR-4715-3p mimic in OE33 or MKN45 cells. (**C**) A schematic drawing shows miR-4715-3p binding sites that were deleted on AURKA 3′UTR (from 508 to 515 bp) to construct a 3′UTR mutant reporter. (**D**,**E**) AURKA 3′UTR luciferase activity reporter assay analysis in OE33 or MKN45 cells with wild-type (D) or mutant (E) AURKA 3′UTR reporters.

We next investigated the possible role of miR-4715-3p in regulating AURKA. As shown in Fig. 2A,B, transient reconstitution of miR-4715-3p (50 picomole for 48 h) led to marked downregulation of AURKA protein and mRNA levels in OE33 and MKN45 cells. We constructed and utilized a luciferase reporter assay, using a reporter containing binding sites of miR-4715-3p on AURKA 3 UTR region, to determine if miR-4715-3p acts directly on AURKA. We cloned the AURKA 3′UTR in a luciferase reporter with or without deleting the binding site of miR-4715-3p on AURKA 3′UTR from 508 to 515 (Fig. 2C). DNA fragments were amplified using PCR and cloned into pLenti-Luc to generate a wild-type and mutant AURKA 3′UTR luciferase reporters. Reconstitution of miR-4715-3p with AURKA wild-type 3′UTR-luc led to a significant reduction in luciferase levels (P < 0.001). On the other hand, transfection of miR-4715-3p with mutant 3′UTR luciferase reporter did not cause changes in the luciferase activity (Fig. 2D,E). These results indicated that AURKA 3′UTR region contains functional binding sites for miR-4715-3p.

### miR-4715-3p reconstitution increasesd polyploidy and decreased spheroid formation

**Figure 3:**
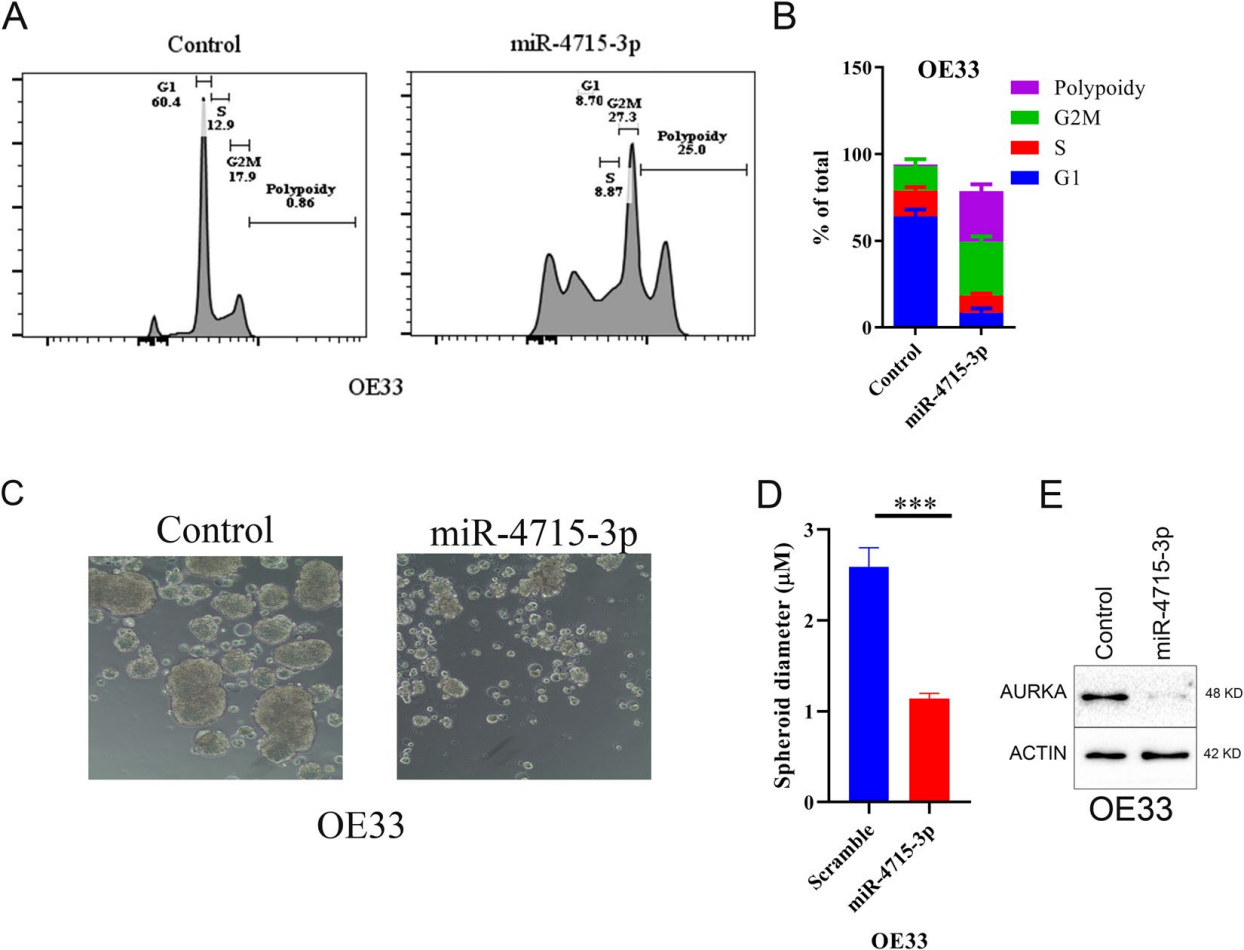
miR-4715-3p suppressed cellular proliferation of OE33 spheroids, enhanced polyploidy, and altered cell cycle progression. (**A**,**B**) OE33 cells were transfected with miR-4715-3p mimic for 48 h, then cell cycle progression was analyzed with flow cytometry. After 48 h, miR-4715-3p reconstitution significantly enhanced polyploidy in OE33 cells. Reconstitution of miR-4715-3p significantly reduced spheroids’ forming ability of OE33 cells (**C**–**E**) Western blot analysis of OE33 spheroids shows significant down regulation of AURKA after miR-4715-3p reconstitution.

As previously reported, AURKA inhibition induced G2/M delays, polyploidy, and cell death^23,24^. Using the OE33 and MKN45 *in vitro* models of UGC, we investigated the effects of miR-4715-3p reconstitution on cell cycle. Transient miR-4715-3p reconstitution for 48 h significantly reduced the percentage of cells in G1‐phase, increased the percentage of cells in G2/M, and increased polyploidy in cancer cells (Fig. 3A,B, and Supplementary Fig. 2) similar to effects of AURKA inhibition. To investigate if miR-4715-3p reconstitution alters spheroid forming ability of MKN45 and OE33 cells, we reconstituted miR-4715-3p in OE33 and MKN45 UGC cells by using lentivirus particles. miR-4715-3p reconstitution significantly decreased the spheroids’ diameter, as compared with control spheroids (Fig. 3C,D, Supplementary Fig. 2C,D, P < 0.001). We confirmed the downregulation of AURKA in spheroids (Fig. 3E, Supplementary Fig. 2E, P < 0.05). Collectively, these results indicated that miR-4715-3p reconstitution in cancer cells caused G/2 M delay, polyploidy, and decreasesd spheroid-forming ability in cancer cells.

### miR-4715-3p reconstitution enhanced cisplatin sensitivity

**Figure 4:**
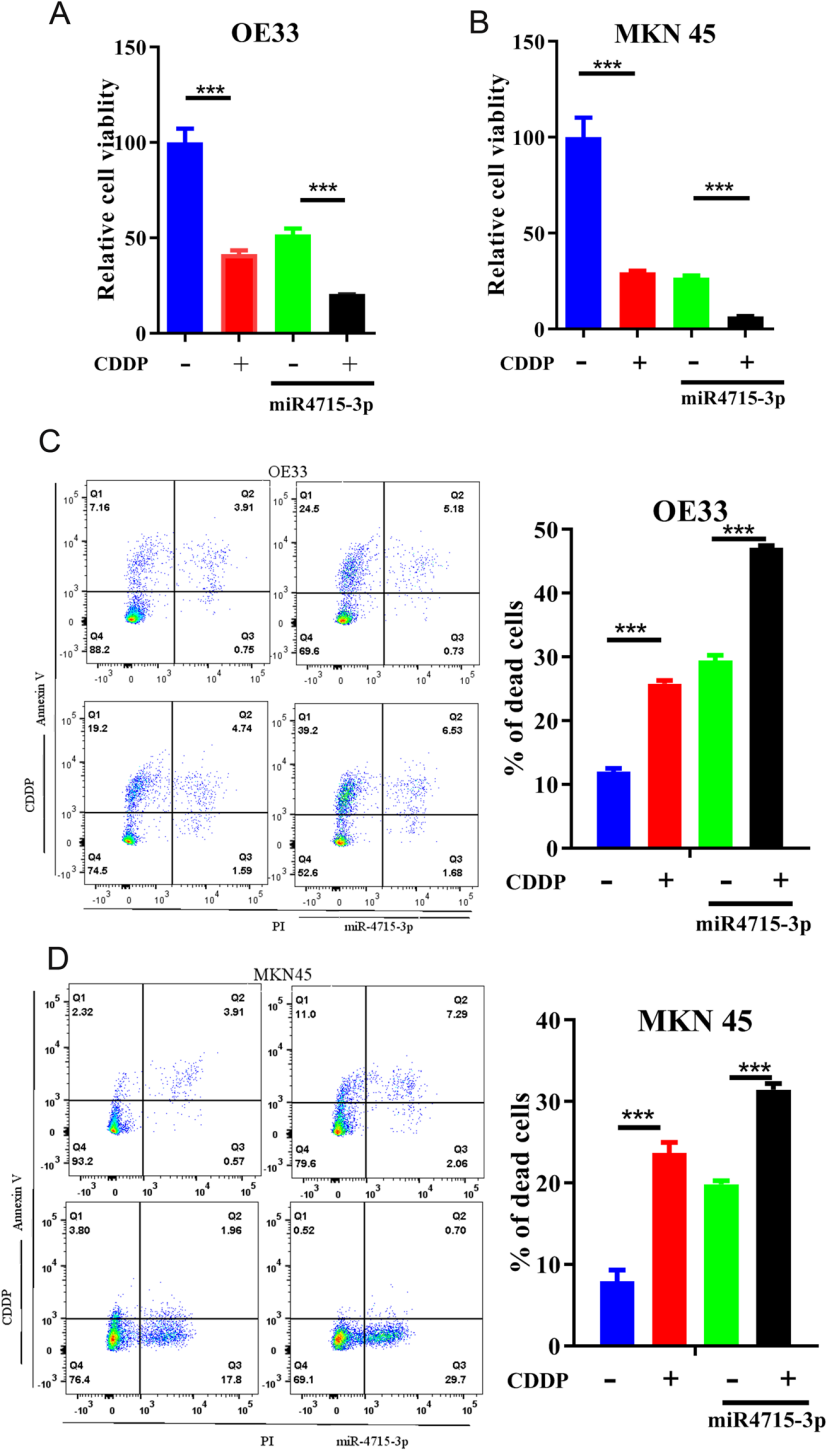
miR-4715-3p overexpression increased sensitivity to cisplatin. ATP-glo cell viability assay analysis of OE33 (**A**) and MKN45 (**B**) cells, following transient transfection with miR-4715-3p. After 48 h, cells were treated with or without cisplatin 5 µM overnight. Flow cytometry analysis of Annexin V positive cells in OE33 and MKN45 cells with miR-4715-3p overexpression or negative control miRNA. (**C**,**D**) left panels show representative flow cytometry profiles and right panels display bar graph of live and apoptotic cells. *p < 0.05, **p < 0.01, ****p < 0.0001.

We investigated the effects of miR-4715-3p reconstitution on UGC cells treated with or without cisplatin (CDDP) a standard chemotherapeutic drug in UGC. Transient miR-4715-3p reconstitution for 72 h led to a significant reduction in the survival of OE33 and MKN45 cancer cells (P < 0.001), comparable to the effects of cisplatin treatment (Fig. 4A,B). Interestingly, miR-4715-3p reconstitution significantly enhanced cisplatin effects (P < 0.001) (Fig. 4A,B). Using Annexin V staining to quantify cell death, we found miR-4715-3p reconstitution significantly promoted cisplatin-induced cell death in OE33 and MKN45 cells (Fig. 4C,D).

### miR-4715-3p reconstitution increased UGC cell death through inhibition of GPX4

**Figure 5:**
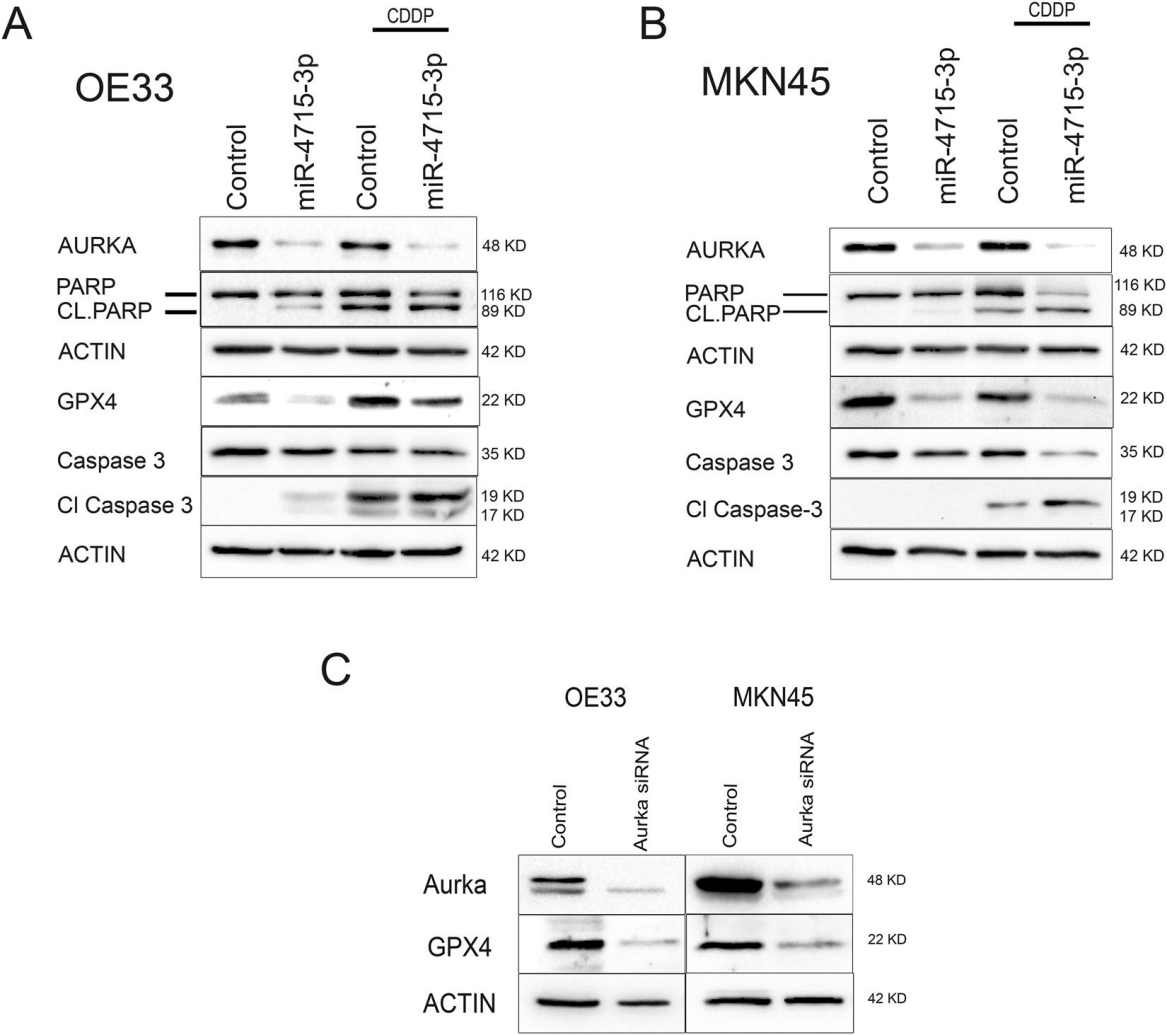
miR-4715-3p overexpression increased cell death through inhibition of GPX4. (**A**,**B**) Western blot analysis of OE33 (**A**) and MKN45 (**B**) cells, following transient transfection with miR-4715-3p. After 48 h, cells were treated with or without cisplatin 5 µM overnight. (**C**) Western blot analysis of OE33 (**A**) and MKN45 (**B**) cells, following transient transfection with AURKA siRNA.

To determine how miR-4715-3p reconstitution induced cell death, we transiently reconstituted miR-4715-3p in OE33, MKN45 and STKM2 cancer cell lines. miR-4715-3p reconstitution led to marked reduction in AURKA levels in the three cell lines and sensitized cells to cisplatin treatment, showing higher levels of cleaved PARP and cleaved caspase 3 (Fig. 5A,B, Supplementary Fig. 3). To determine the type of cell death, we investigated the effects of miR-4715-3p on GPX4 levels (inhibitor of ferroptosis)^25,26^. Interestingly, miR-4715-3p reconstitution markedly decreased protein expression of GPX4, suggesting the occurrence of ferroptosis. To determine if miR-4715-3p reconstitution decreased GPX4 through AURKA-dependent mechanism, we knocked down AURKA using siRNA. AURKA knockdown markedly decreased GPX4 expression, similar to the miR-4715-3p reconstitution results (Fig. 5C). These findings identified AURKA as an inhibitor of ferroptosis where its knockdown or downregulation with miR-4715-3p reconstitution promoted ferroptotic cell death via inhibition of GPX4.

### miR-4715-3p methylation mediated miR-4715-3p downregulation

**Figure 6:**
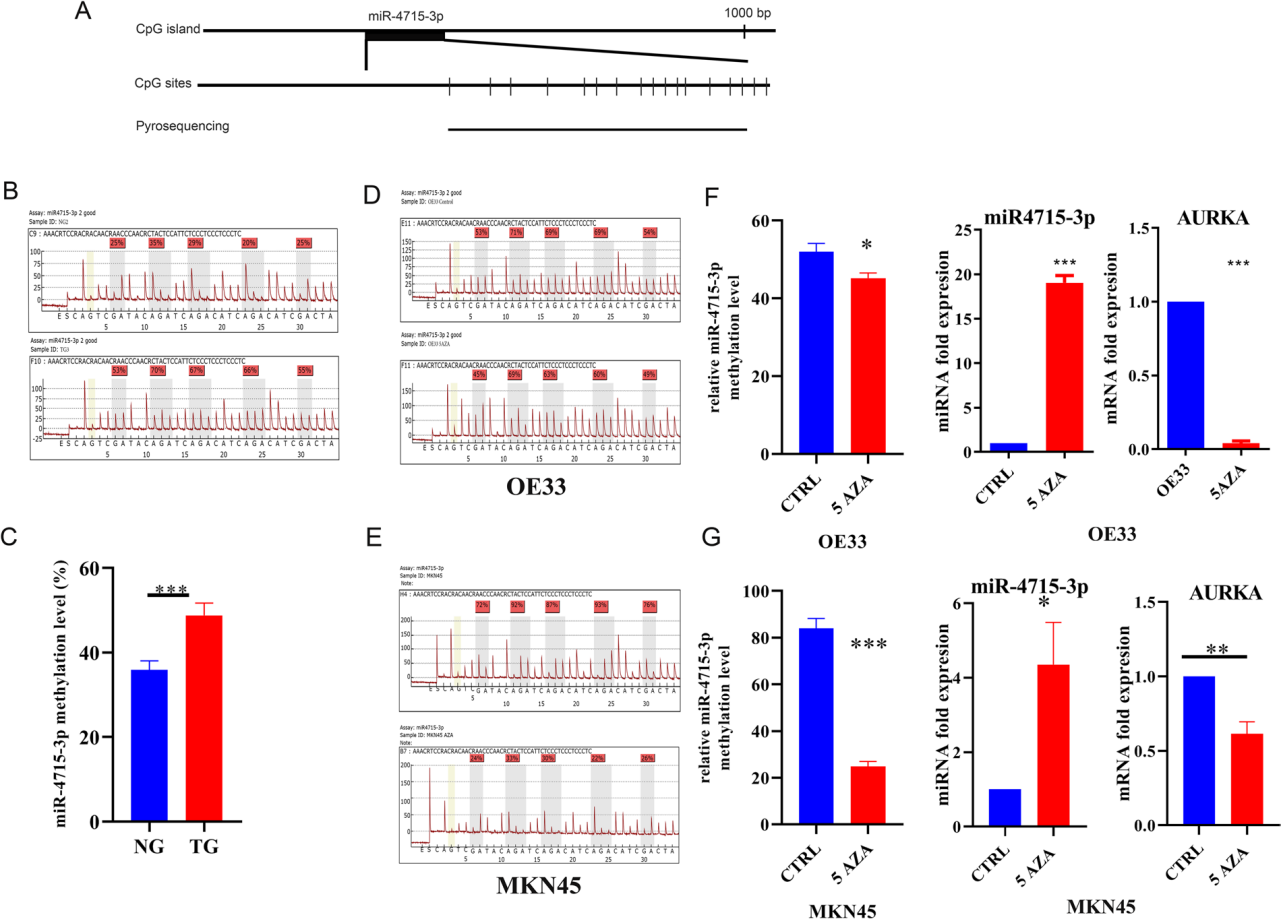
miR-4715-3p is hypermethylated in UGCs samples. (**A**) A schematic drawing shows the CpG sites in miR-4715-3p and pyrosequencing assay location. Each vertical bar represents a CpG site. DNA methylation levels of 5 CpG sites in the 1,000 bp upstream of miR-4715-3p was quantified by pyrosequencing. (**B**) Representative pyrosequencing profiles of normal and gastric cancer tissue samples. (**C**) Quantification of miR-4715-3p methylation in normal and gastric cancer tissue samples (**D**,**E**) Representative pyrosequencing profiles of OE33 and MKN45 cells. (**F**,**G**) AURKA mRNA and miR-4715-3p expression levels in OE33 and MKN45 cells treated with 5-Aza-2′-deoxycytidine (Decitabine), 5 µM for 72 h.

DNA methylation is an important epigenetic mechanism that controls gene expression. Using human genome browser (https://genome.ucsc.edu/), we analyzed 1,000 bp upstream of miR-4715-3p, as a potential candidate promoter region, and identified multiple CpG nucleotides (Fig. 6A). Therefore, we investigated CpG methylation as a potential regulatory mechanism that controls miR-4715-3p expression. Using quantitative pyrosequencing on normal and tumor human tissue samples, we detected increased methylation levels in cancer samples, as compared with normal samples (Fig. 6B,C). We analyzed methylation pattern of the same region in OE33, MKN45 and STKM2 cancer cells treated with or without 5-Aza-2′deoxycytidine (5-aza), a DNA methyl transferase inhibitor. This treatment led to a significant reduction in CpG nucleotide methylation levels (P < 0.001) (Fig. 6D,E, Supplementary Fig. 4A–C) with a significant increase in miR-4715-3p and decrease in AURKA and GPX4 expression levels, as compared with no treatment control (Fig. 6F,G, Supplementary Fig. 4D,E). These results suggested that epigenetic DNA methylation played an important role in regulating miR-4715-3p, AURKA and GPX4 expression levels in UGC cells.

## Discussion

AURKA is a serine threonine kinase that plays a major role in mitotic progression in normal cells^27^. Several recent studies have shown frequent overexpression of AURKA in several cancer types^28–32^, including gastric and esophageal cancers^33–35^. Molecular and functional studies identified several oncogenic functions of constitutively overexpressed AURKA in cancer. AURKA can inhibit tumor suppressor genes such as p53^23^ and p73^36,37^, and activate β-catenin^38^, NF-kB^39^, and cap-dependent translation of oncogenes^40^.

Although genomic amplifications of AURKA gene have been described in cancer^41–45^, these do not fully explain the prevalent frequency of high levels of AURKA protein. In this study, we investigated epigenetic regulation of AURKA in UGC. We previously reported miRNA-seq and identified frequent alterations of miRNAs in human and mouse gastric cancers^22^. Among the downregulated miRNAs, predicted to bind AURKA 3″ UTR, miR-4715-3p was significantly downregulated in human and mouse gastric cancers. Our molecular analysis suggested that, miR-4715-3p can bind to AURKA 3′UTR to negatively regulate its levels in UGC. This finding provided a plausible explanation for the frequent overexpression of AURKA in UGCs, a finding that may be applicable to several other cancer types. However, this must be confirmed as miRNAs expression and functions are known to be tissue and context specific^46^. Of note, our analysis indicated that AURKA overexpression, as well as miR-4715-3p downregulation, correlated with an overall poor patients’ survival rate.

Inhibition of AURKA is known to induce G2/M delays with increase in polyploidy^34,47^. Our analysis demonstrated similar functions following reconstitution of miR-4715-3p. This finding confirmed the functional validity of miR-4715-3p in regulating AURKA levels. We also detected increased sensitivity to cisplatin under the same conditions, a result that was further confirmed in spheroid cell models. Our findings agree with earlier results showing a synergistic effect of alisertib (AURKA inhibitor) and cisplatin in cancer cells^21,48^.

Protection against ferroptosis, a form of cell death, is recognized as an important molecular mechanism that promotes drug resistance in cancers^49,50^. Glutathione peroxidase-4 (GPX4) is a key regulator of ferroptosis^51,52^. For example, inhibition of cysteine dioxygenase 1 (CDO1) prevents ferroptotic cell death through upregulation of GPX4^53^. Inhibition of AURKA by genetic knockdown or via reconstitution of miR-4715-3p led to a marked reduction of GPX4. These results identified a novel function of AURKA in regulating GPX4 levels, providing a link between overexpression of AURKA and protection against ferroptosis in cancer cells.

Aberrant DNA methylation in human cancers disrupt expression of several tumor suppressor genes and signaling pathways during tumorigenesis and cancer progression^54^. For example, promoter hypermethylation induces downregulation of E-cadherin and chromodomain helicase DNA binding protein 5 (CHD5) promoting gastric tumorigenesis^55,56^. We detected high methylation levels of CpG sites in an upstream predicted promoter of miR-4715-3p in tumor samples and cancer cell lines. The use of 5-Aza-2′deoxycytidine (5 AZA) a demethylation agent, increased expression miR-4715-3p with concomitant decrease in AURKA levels. This finding suggests DNA hypermethylation as an important cause for miR-4715-3p downregulation in UGCs.

In conclusion, this study highlights a novel epigenetic mechanism that mediates AURKA overexpression in cancer. Silencing of miR-4715-3p by DNA methylation promotes high expression levels of AURKA in UGCs. Inhibition of AURKA or reconstitution of miR-4715-3p induced cell death and inhibited GPX4, suggesting a link between high levels of AURKA and protection against ferroptosis.

## Material and Methods

### Tissue samples

All de-identified gastric tissue samples were obtained following written informed consents in accordance with approval from the Institutional Review Board‐approved protocols at the University of Miami. The use of archival de-identified human tissues samples was approved as non-human subjects in accordance with the University of Miami’s institutional review board. For DNA, mRNA, and miRNA analyses, 72 de-identified archival human gastric tissue samples (49 non-tumor normal stomach and 33 gastric cancer samples) were used. All adenocarcinomas were classified according to the recent guidelines of the International Union against Cancer TNM classification system.

### Patients’ survival data analysis

We analyzed AURKA and miR-4715-3p relationship to overall survival of 876 and 436 gastric cancer patients, respectively. The data was obtained from an online database (http://kmplot.com/analysis/)^57,58^. Kaplan-Meier survival analysis was performed using SPSS, version 24 (IBM, Armonk, NY).

### Quantitative real-time RT-PCR

Total RNA was prepared from cell lines using the RNeasy Mini Kit (Qiagen, Germantown, MD, USA). Total RNA (1 μg) was reverse transcribed to miRNA cDNA using the TaqMan MicroRNA Reverse Transcription Kit (ThermoFisher Scientific) and E. coli Poly A polymerase (New England Biolabs). qRT-PCR was performed using a Bio-Rad CFX Connect Real-time System (Bio-Rad) with the threshold cycle number determined by the Bio-Rad CFX manager software V.3.0. mRNA expression was normalized to HPRT (29) and miRNA expression was normalized to the average miR-140-3p and miR-101-3p^21^. The following primers were used for PCR analysis: miR-4715-3p 5′-GTGCCACCTTAACTGCAGCCAT-′3 (sense) and 5′-GCGAGCACAGAATTAATACGAC-′3 (antisense) miR-101-3p 5′-TACAGTACTGTGATAACTGAA-′3 (sense) 5′-GCGAGCACAGAATTAATACGAC-′3 (antisense) miR-140-3p 5′-TACCACAGGGTAGAACCACGG-′3 (sense) 5′-GCGAGCACAGAATTAATACGAC-′3 (antisense), AURKA 5′-AGTTGGAGGTCCAAAACGTG-′3 (sense) 5′-TCCAAGTGGTGC ATATTCCA-′3 (antisense) HPRT1, 5′- TTGGAAAGGGTGTTTATTCCTCA -3′ (sense) 5′-TCCAGCAGGTCAGCAAAGAA -3′ (antisense).

### Cell culture and reagents

OE33, a human esophageal adenocarcinoma cell line, was a kind gift from Dr. David Beer (University of Michigan, Ann Arbor, MI, USA). The MKN45 gastric cancer cell line was obtained from RIKEN BioResource Center (Tsukuba, Japan). STKM2 gastric cancer cell line^59,60^ was a kind gift from Dr. Alexander Zaika (University of Miami Miller School of Medicine). Cells were maintained in RPMI 1640 (Cellgro, CA, USA) supplemented with 10% fetal bovine serum (FBS, Gemini-Bio-Products, CA, USA) 100 units/ml penicillin, 100 units/ml streptomycin, and 2 mM glutamine at 37 °C in a humidified atmosphere with 5% CO2, and were in the logarithmic growth phase upon initiation of experiments. MNK45 cells were passaged for ≤3 months before fresh cells were obtained from frozen early-passage stocks, received from the indicated sources. All cell lines were authenticated by Genetica DNA Laboratories (Genetica DNA Laboratories). Mycoplasma was negative in all cells, using the PCR method (Mycoplasma Detection Kit, Southern Biotech). Demethylation was induced with 5-Aza-2′ deoxycytidine (5-Aza; Selleckchem) treatment at a concentration of 5 μmol/L. Cells were incubated for 72 h with 5-Aza with replacement of the culture media with fresh media containing 5-Aza every 24 h. Antibodies against AURKA, PARP, cleaved PARP, caspase-3, cleaved caspase-3, and β-Actin were purchased from Cell Signaling Technology (Beverly, MA). The antibody against GPX4 was purchased from R&D Systems, Inc. (Minneapolis, MN).

### DNA bisulfite treatment and quantitative pyrosequencing analysis

OE33, STKM2 and MKN45 cells were cultured with 5 μmol/L 5-Aza, as described above. DNA was purified by DNeasy Blood & Tissue Kits (Qiagen). The DNA was bisulfite converted using an EZ DNA Methylation Gold Kit (Zymo Research) according to the manufacturer’s protocol. Specific bisulfite PCR primers and sequencing primers for pyrosequencing were designed using the PSQ assay design software (Qiagen). The assay is to analyze five CpG dinucleotide sites in a CpG island 1,000 bp upstream of miR-4715-3p. The following primers were used: forward 5′-Biotin-GGGAGGGAGAATGGAGTAG-′3, reverse 5′-ACCCTAAAAACCAACCCACAAA-′3, sequencing primer 5′-AATTTTAAAAAACTAAAACTATATA-′3. A 20 ng aliquot of modified DNA was amplified by PCR using the Platinum PCR SuperMix High Fidelity Enzyme Mix (Invitrogen). PCR products were checked by gel electrophoresis to confirm the size of the product and to rule out the formation of primer dimers. The specific PCR products were analyzed using a Biotage PyroMark MD System (Qiagen) according to the manufacturer’s protocol, using PyroMark Gold Reagents (Qiagen). Using normal and positive control samples, DNA methylation was calculated using Pyromark software^61^.

### Western blot

Cells were scraped on ice, centrifuged, and pellets were re-suspended in RIPA lysis buffer (Santa Cruz). Cell lysates were placed on ice. Protein concentration was determined using BCA (ThermoScientific, Waltham, PA). Equal amounts of protein were subjected to SDS/PAGE and transferred onto nitrocellulose membranes using the semi-dry transfer protocol (Bio-Rad Laboratories, Hercules, CA). After transfer, membranes were probed with each respective primary antibody overnight at 4 °C. Following incubation, the membranes were probed with HRP-conjugated secondary antibodies (Bio-Rad). Protein bands were visualized using the commercial Immobile Western Chemiluminescent HRP Substrate Kit (Millipore, Billerica, MA). Images of immunoblots were obtained using the ChemiDoc XRS+ system (Bio-Rad).

### CellTiter-Glo luminescence assay

Cells were seeded at 2,000 cells/well in a 96-well plate. Following transient transfection with miR-4715-3p with or without cisplatin treatment, cells were seeded at 2,000 cells/well in a 96-well plate. Cells were treated with 5 uM cisplatin treatment in 5% FBS-RPMI 1640. After 3 days, cell viability was calculated using the CellTiter-Glo Luminescence Assay (Promega). GraphPad Prism 9 was used to obtain the curves (GraphPad Software, Inc., San Diego, CA, USA). Three independent experiments were performed to generate data.

### miR-4715-3p reconstitution and AURKA silencing by small interfering RNA (siRNA)

OE33, MKN45 and STKM2 cells were seeded at 60–70% confluency in 10% FBS RPMI 1640 in 6 well plates. Validated AURKA siRNA (Invitrogen) was used to transiently silence AURKA, using LipoJet reagent (SignaGen). Western blot analysis was performed to validate AURKA knockdown.

### Lentiviral infection

OE33, MKN45 cells were transfected with LentimiRa-GFP-has-miR-4715-3p lentiviral plasmids (Applied Biological Materials, Richmond, BC, Canada) using LipoJet (SignaGen Laboratories (Gaithersburg, MD). Production of lentivirus particles was performed using 2nd Generation Packaging System Mix vector (Applied Biological Materials) into 293FT cells. Selection of transfected cells was performed by treating cells with 1 μg/ml puromycin for 48 h.

### Flow cytometry

For cell cycle analysis, OE33 and MKN45 cells were transiently transfected with miR-4715-3p for 48 h. Cells were harvested and fixed in 70% ethanol. Cells were stained with Propidium Iodide (PI) solution (PI 50 μg/ml and RNase 1 μg/ml dissolved Phosphate Buffered Saline) for 15–30 min. Finally, we analysed the cell cycle using BD LSR III Flow Cytometer (BD Biosciences, San Jose, CA). The data was processed by using the Flowjo software. The FITC Annexin V kit (BD Biosciences) was used to analyze cell death, following miR-4715-3p overexpression. OE33 and MKN45 cells were transfected with 50 picomole miR-4715-3p mimic and/or control (Applied Biological Materials), using the LipoJet transfection reagent (SignaGen Laboratories, Rockville, MD). After 48 h, cells were treated with cisplatin for 24 h. The FITC Annexin V and PI staining were performed according to the manufacturer’s instructions. The Flow Cytometry Shared Resources at Sylvester Comprehensive Cancer Center was used for analysis of data.

### Luciferase reporter assay

To confirm the binding of miR-4715-3p to the 3′ UTR regions of AURKA, OE33 and MKN45 cells were transfected with miR-4715-3p mimic or control. After 24 h, cells were transfected with 250 ng AURKA 3′-UTR Luciferase/GFP reporter vector, purchased from Abmgood (Applied Biological Materials, Richmond, BC, Canada) and 60 ng of ubiquitin promoter Renilla luciferase plasmid (control), using Polyjet (SignaGen Laboratories). We collected cells for analysis after 48 h. The Firefly luciferase activities were normalized to Renilla luciferase levels. The miR-4715-3p binding site in AURKA 3′ UTR Luciferase/GFP reporter vectors were deleted using the QuikChange Lightning Site-Directed Mutagenesis Kit from Agilent Technologies (Santa Clara, CA). We confirmed the deletion of miR-4715-3p binding site by DNA sequencing (Supplementary Fig. 1).

### Statistical analysis

GraphPad Prism 8 software (GraphPad Software Inc.) was used for analysis of statistical significance. We applied One-way ANOVA Test to assess the difference between the treatment and control groups. We presented the data as means +/− standard error of mean (SEM). Two-tailed Student’s test was used to analyze the correlation between two parameters. A *P* value ≤ 0.05 was considered significant.

## Supplementary information

Supplementary Figures

## References

[1] Jemal A (2011). Global cancer statistics. CA: a cancer journal for clinicians.

[2] Siegel RL, Miller KD, Jemal A (2019). Cancer statistics, 2019. CA: a cancer journal for clinicians.

[3] Calin GA, Croce CM (2006). MicroRNA signatures in human cancers. Nature reviews. Cancer.

[4] Gulyaeva LF, Kushlinskiy NE (2016). Regulatory mechanisms of microRNA expression. Journal of translational medicine.

[5] Bartel DP (2004). MicroRNAs: genomics, biogenesis, mechanism, and function. Cell.

[6] Qin J (2015). Downregulation of microRNA-132 by DNA hypermethylation is associated with cell invasion in colorectal cancer. Onco Targets Ther.

[7] Deneberg S (2014). microRNA-34b/c on chromosome 11q23 is aberrantly methylated in chronic lymphocytic leukemia. Epigenetics.

[8] Augoff Katarzyna, McCue Brian, Plow Edward F, Sossey-Alaoui Khalid (2012). miR-31 and its host gene lncRNA LOC554202 are regulated by promoter hypermethylation in triple-negative breast cancer. Molecular Cancer.

[9] Katsha A, Belkhiri A, Goff L, El-Rifai W (2015). Aurora kinase A in gastrointestinal cancers: time to target. Mol Cancer.

[10] Peng D (2017). Integrated molecular analysis reveals complex interactions between genomic and epigenomic alterations in esophageal adenocarcinomas. Scientific reports.

[11] Comprehensive molecular characterization of gastric adenocarcinoma. *Nature* **513**, 202–209, 10.1038/nature13480 (2014). 10.1038/nature13480 PMC4170219 25079317

[12] Integrated genomic characterization of oesophageal carcinoma, *Nature* **541**, 169–175, 10.1038/nature20805 (2017). 10.1038/nature20805 PMC5651175 28052061

[13] Aradottir M (2015). Aurora A is a prognostic marker for breast cancer arising in BRCA2 mutation carriers. The journal of pathology. Clinical research.

[14] Gorgun G (2010). A novel Aurora-A kinase inhibitor MLN8237 induces cytotoxicity and cell-cycle arrest in multiple myeloma. Blood.

[15] Otto T, Sicinski P (2017). Cell cycle proteins as promising targets in cancer therapy. Nature reviews. Cancer.

[16] Bischoff JR (1998). A homologue of Drosophila aurora kinase is oncogenic and amplified in human colorectal cancers. Embo j.

[17] Zheng F (2016). Nuclear AURKA acquires kinase-independent transactivating function to enhance breast cancer stem cell phenotype. Nature communications.

[18] Dauch D (2016). A MYC-aurora kinase A protein complex represents an actionable drug target in p53-altered liver cancer. Nature medicine.

[19] Richards MW (2016). Structural basis of N-Myc binding by Aurora-A and its destabilization by kinase inhibitors. Proceedings of the National Academy of Sciences of the United States of America.

[20] Katsha A (2017). Activation of EIF4E by Aurora Kinase A Depicts a Novel Druggable Axis in Everolimus-Resistant Cancer Cells. Clinical cancer research: an official journal of the American Association for Cancer Research.

[21] Wang L (2017). Cisplatin-resistant cancer cells are sensitive to Aurora kinase A inhibition by alisertib. Mol Oncol.

[22] Chen Zheng, Li Zheng, Soutto Mohammed, Wang Weizhi, Piazuelo M. Blanca, Zhu Shoumin, Guo Yan, Maturana Maria J., Corvalan Alejandro H., Chen Xi, Xu Zekuan, El-Rifai Wael M. (2019). Integrated Analysis of Mouse and Human Gastric Neoplasms Identifies Conserved microRNA Networks in Gastric Carcinogenesis. Gastroenterology.

[23] Sehdev V (2014). HDM2 regulation by AURKA promotes cell survival in gastric cancer. Clinical cancer research: an official journal of the American Association for Cancer Research.

[24] Zhu Q (2017). Inhibition of Aurora A Kinase by Alisertib Induces Autophagy and Cell Cycle Arrest and Increases Chemosensitivity in Human Hepatocellular Carcinoma HepG2 Cells. Current cancer drug targets.

[25] Wu J (2019). Intercellular interaction dictates cancer cell ferroptosis via NF2–YAP signalling. Nature.

[26] Yang WS (2014). Regulation of ferroptotic cancer cell death by GPX4. Cell.

[27] Li M, Gao K, Chu L, Zheng J, Yang J (2018). The role of Aurora-A in cancer stem cells. The international journal of biochemistry & cell biology.

[28] Cammareri P (2010). Aurora-a is essential for the tumorigenic capacity and chemoresistance of colorectal cancer stem cells. Cancer research.

[29] Tanaka T (1999). Centrosomal kinase AIK1 is overexpressed in invasive ductal carcinoma of the breast. Cancer research.

[30] Li D (2003). Overexpression of oncogenic STK15/BTAK/Aurora A kinase in human pancreatic cancer. Clinical cancer research: an official journal of the American Association for Cancer Research.

[31] Kamada K (2004). Amplification/overexpression of Aurora-A in human gastric carcinoma: potential role in differentiated type gastric carcinogenesis. Oncology reports.

[32] Yang SB (2007). Amplification and overexpression of Aurora-A in esophageal squamous cell carcinoma. Oncology reports.

[33] Mesic A, Rogar M, Hudler P, Juvan R, Komel R (2016). Association of the AURKA and AURKC gene polymorphisms with an increased risk of gastric cancer. IUBMB life.

[34] Liu X (2016). AURKA induces EMT by regulating histone modification through Wnt/beta-catenin and PI3K/Akt signaling pathway in gastric cancer. Oncotarget.

[35] Kamran M (2017). Aurora kinase A regulates Survivin stability through targeting FBXL7 in gastric cancer drug resistance and prognosis. Oncogenesis.

[36] Dar AA, Belkhiri A, Ecsedy J, Zaika A, El-Rifai W (2008). Aurora kinase A inhibition leads to p73-dependent apoptosis in p53-deficient cancer cells. Cancer research.

[37] Katayama H (2012). Aurora kinase-A inactivates DNA damage-induced apoptosis and spindle assembly checkpoint response functions of p73. Cancer Cell.

[38] Dar AA, Belkhiri A, El-Rifai W (2009). The aurora kinase A regulates GSK-3beta in gastric cancer cells. Oncogene.

[39] Katsha A (2013). Aurora kinase A promotes inflammation and tumorigenesis in mice and human gastric neoplasia. Gastroenterology.

[40] Kochupurakkal BS, Iglehart JD (2016). Identification of genes responsible for RelA-dependent proliferation arrest in human mammary epithelial cells conditionally expressing RelA. Genomics data.

[41] Koh HM (2017). Aurora Kinase A Is a Prognostic Marker in Colorectal Adenocarcinoma. J Pathol Transl Med.

[42] Puig-Butille JA (2017). AURKA Overexpression Is Driven by FOXM1 and MAPK/ERK Activation in Melanoma Cells Harboring BRAF or NRAS Mutations: Impact on Melanoma Prognosis and Therapy. J Invest Dermatol.

[43] Banck MS (2013). The genomic landscape of small intestine neuroendocrine tumors. J Clin Invest.

[44] Li X (2018). Genomic analysis of liver cancer unveils novel driver genes and distinct prognostic features. Theranostics.

[45] Ju H (2006). Functional polymorphism 57Val>Ile of aurora kinase A associated with increased risk of gastric cancer progression. Cancer letters.

[46] Macfarlane LA, Murphy PR (2010). MicroRNA: Biogenesis, Function and Role in Cancer. Current genomics.

[47] Sehdev V (2013). The combination of alisertib, an investigational Aurora kinase A inhibitor, and docetaxel promotes cell death and reduces tumor growth in preclinical cell models of upper gastrointestinal adenocarcinomas. Cancer.

[48] Xu J (2014). Aurora-A contributes to cisplatin resistance and lymphatic metastasis in non-small cell lung cancer and predicts poor prognosis. Journal of translational medicine.

[49] Xie Y (2017). The Tumor Suppressor p53 Limits Ferroptosis by Blocking DPP4 Activity. Cell reports.

[50] Ma S, Henson ES, Chen Y, Gibson SB (2016). Ferroptosis is induced following siramesine and lapatinib treatment of breast cancer cells. Cell death & disease.

[51] Friedmann Angeli JP (2014). Inactivation of the ferroptosis regulator Gpx4 triggers acute renal failure in mice. Nature cell biology.

[52] Imai H, Matsuoka M, Kumagai T, Sakamoto T, Koumura T (2017). Lipid Peroxidation-Dependent Cell Death Regulated by GPx4 and Ferroptosis. Current topics in microbiology and immunology.

[53] Hao S (2017). Cysteine Dioxygenase 1 Mediates Erastin-Induced Ferroptosis in Human Gastric Cancer Cells. Neoplasia (New York, N.Y.).

[54] Chen G, Lu L, Liu C, Shan L, Yuan D (2015). MicroRNA-377 suppresses cell proliferation and invasion by inhibiting TIAM1 expression in hepatocellular carcinoma. PloS one.

[55] Abukiwan A (2019). Dexamethasone-induced inhibition of miR-132 via methylation promotes TGF-beta-driven progression of pancreatic cancer. International journal of oncology.

[56] Tamura G (2000). E-Cadherin gene promoter hypermethylation in primary human gastric carcinomas. Journal of the National Cancer Institute.

[57] Szasz AM (2016). Cross-validation of survival associated biomarkers in gastric cancer using transcriptomic data of 1,065 patients. Oncotarget.

[58] Nagy A, Lanczky A, Menyhart O, Gyorffy B (2018). Validation of miRNA prognostic power in hepatocellular carcinoma using expression data of independent datasets. Scientific reports.

[59] Chen Z (2019). Integrated Analysis of Mouse and Human Gastric Neoplasms Identifies Conserved microRNA Networks in Gastric Carcinogenesis. Gastroenterology.

[60] Nie XC (2013). COL4A3 expression correlates with pathogenesis, pathologic behaviors, and prognosis of gastric carcinomas. Hum Pathol.

[61] Peng DF (2009). DNA hypermethylation regulates the expression of members of the Mu-class glutathione S-transferases and glutathione peroxidases in Barrett’s adenocarcinoma. Gut.

