# Supplementary material for: Epigenetic regulation of AURKA by miR-4715-3p in upper gastrointestinal cancers
Source: Sci Rep. 2019 Nov 18;9:16970. doi: 10.1038/s41598-019-53174-6 (PMC6861278; doi:10.1038/s41598-019-53174-6)
Supplement: Supplementary file 1 — Supplementary Figures [file 41598_2019_53174_MOESM1_ESM.pdf]

Epigenetic regulation of AURKA by miR-4715-3p in upper gastrointestinal cancers

Ahmed Gomaa[1], Dunfa Peng[1], Zheng Chen[1, 2], Mohammed Soutto [1, 2], Khaled Abouelezz[3]

Alejandro Corvalan[4], Wael El-Rifai [1, 2]

[1] Department of Surgery, University of Miami, Miami, Florida, USA.

[2] Department of Veterans Affairs, Miami Healthcare System, Miami, Florida, USA.

[3] Department of Medicine, University of Miami, Miami, Florida, USA.

[4] Advanced Center for Chronic Disease, School of Medicine, Pontificia Universidad Catolica de Chile, Portugal, Santiago, Chile

Supplementary Figure 1

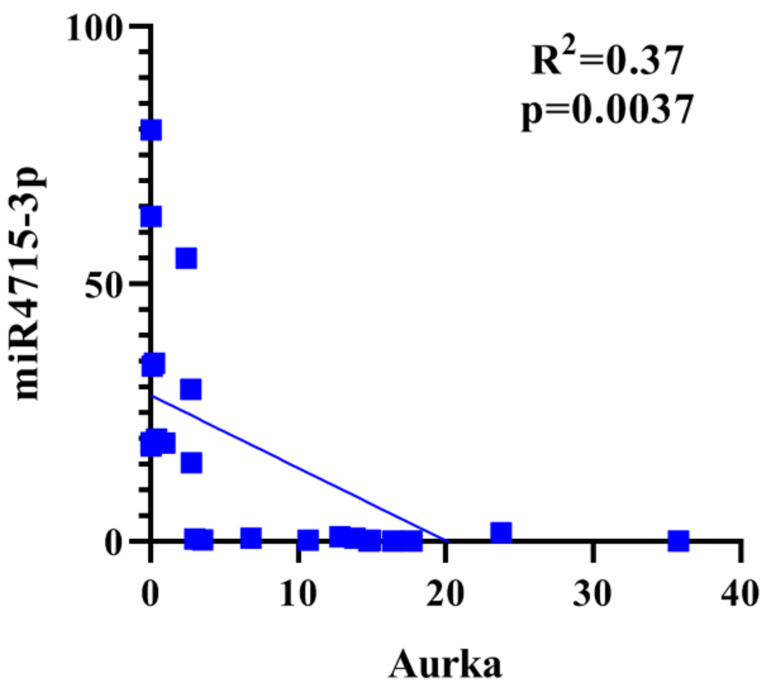

Supplementary Figure 1. Inverse correlation of expression AUKRA and miR4715-3p. Normal gastric and tumor human tissues were analyzed for the level of AURKA and miR-47-15-3p using qPCR.

Supplementary Figure 2

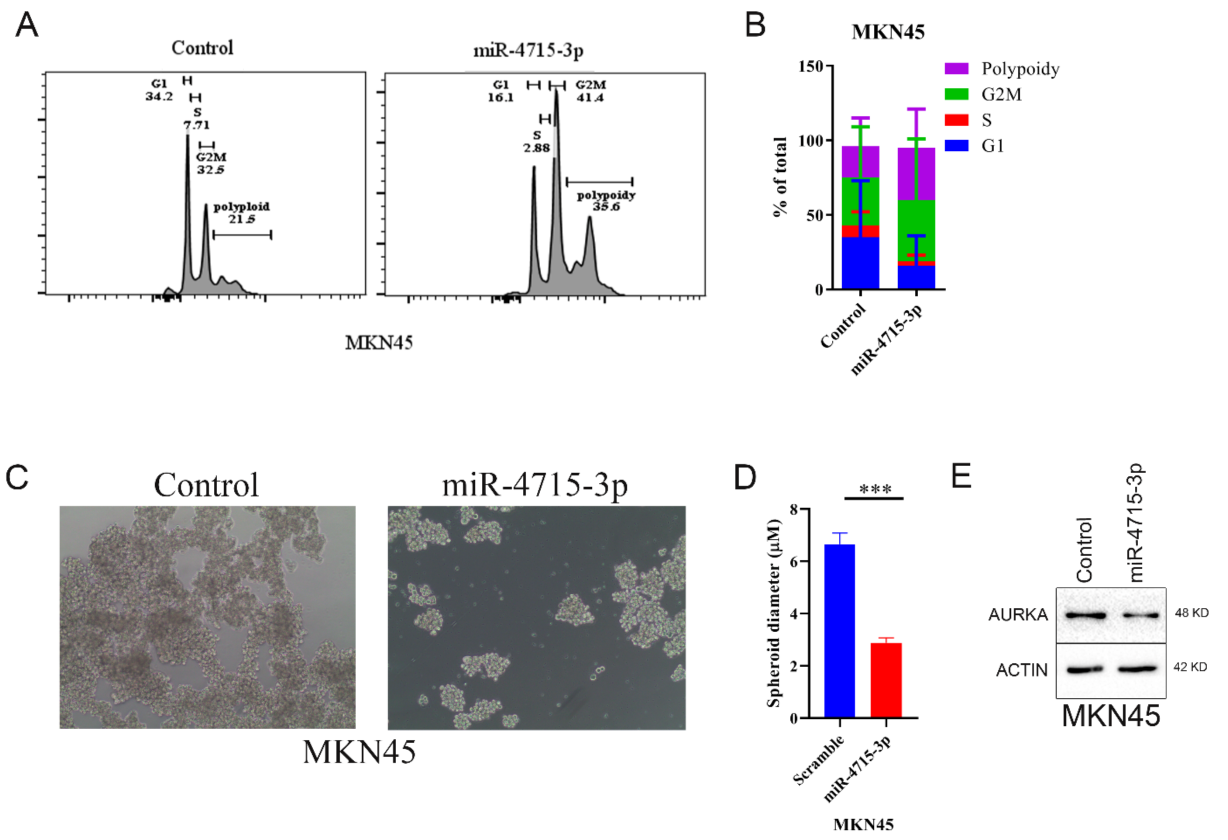

Supplementary Figure 2. miR-4715-3p suppresses cellular proliferation, and spheroid growth, enhances polyploidy, and alters cell cycle progression. (A & B) MKN45 cells were transfected with miR-4715-3p mimic 48h, and cell cycle progression was analyzed by flow cytometry. After 48h, miR-4715-3p reconstitution significantly enhanced polyploidy in MKN45 cells. **(C & D)** Reconstitution of miR-4715-3p was established by using lentiviral infection followed by establishing spheroid cultures. **(E)** Western blot analysis of MKN45 spheroids shows significant downregulation of AURKA after miR-4715-3p reconstitution.

Supplementary Figure 3

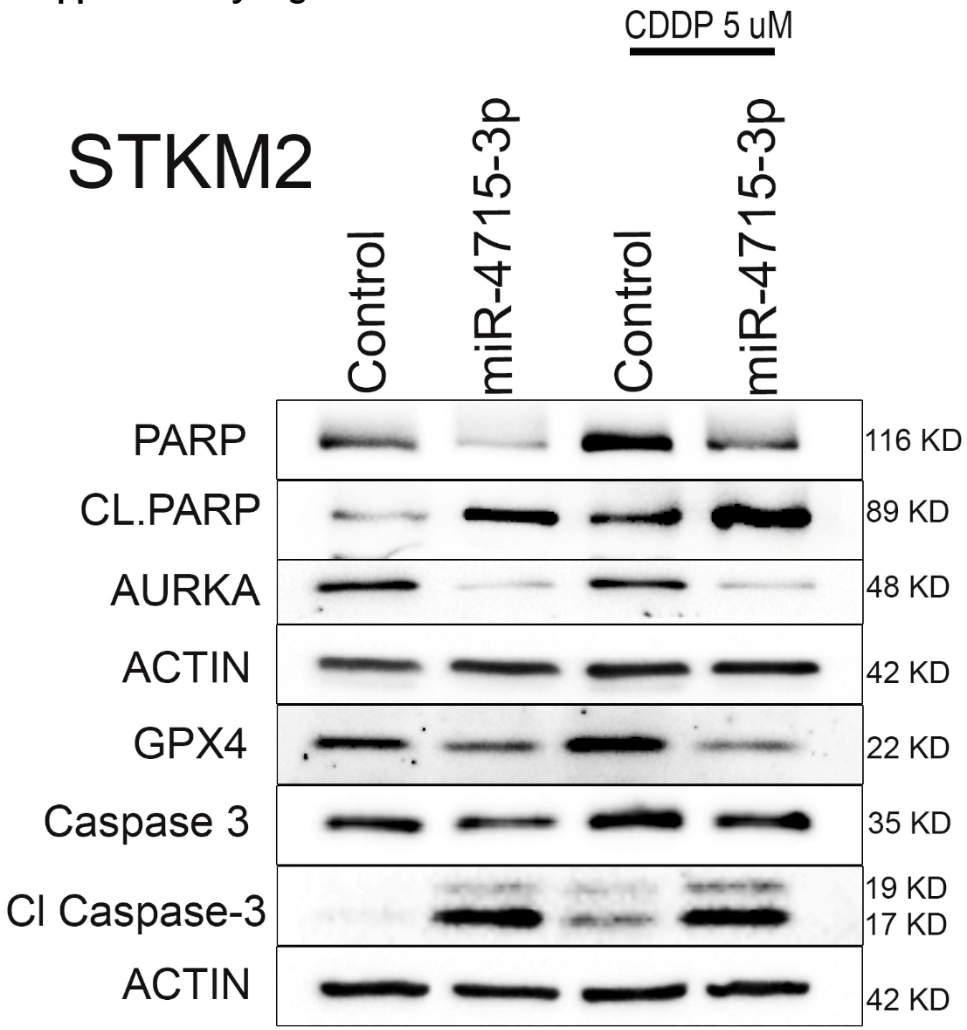

Supplementary Figure 3. miR-4715-3p overexpression increases gastric cancer cell line, STKM2, death through inhibition of GPX4. STKM2 cells were transiently transfected with miR-4715-3p. After 48 h, cells were treated with or without Cisplatin 5 μM 18h

Supplementary Figure 4

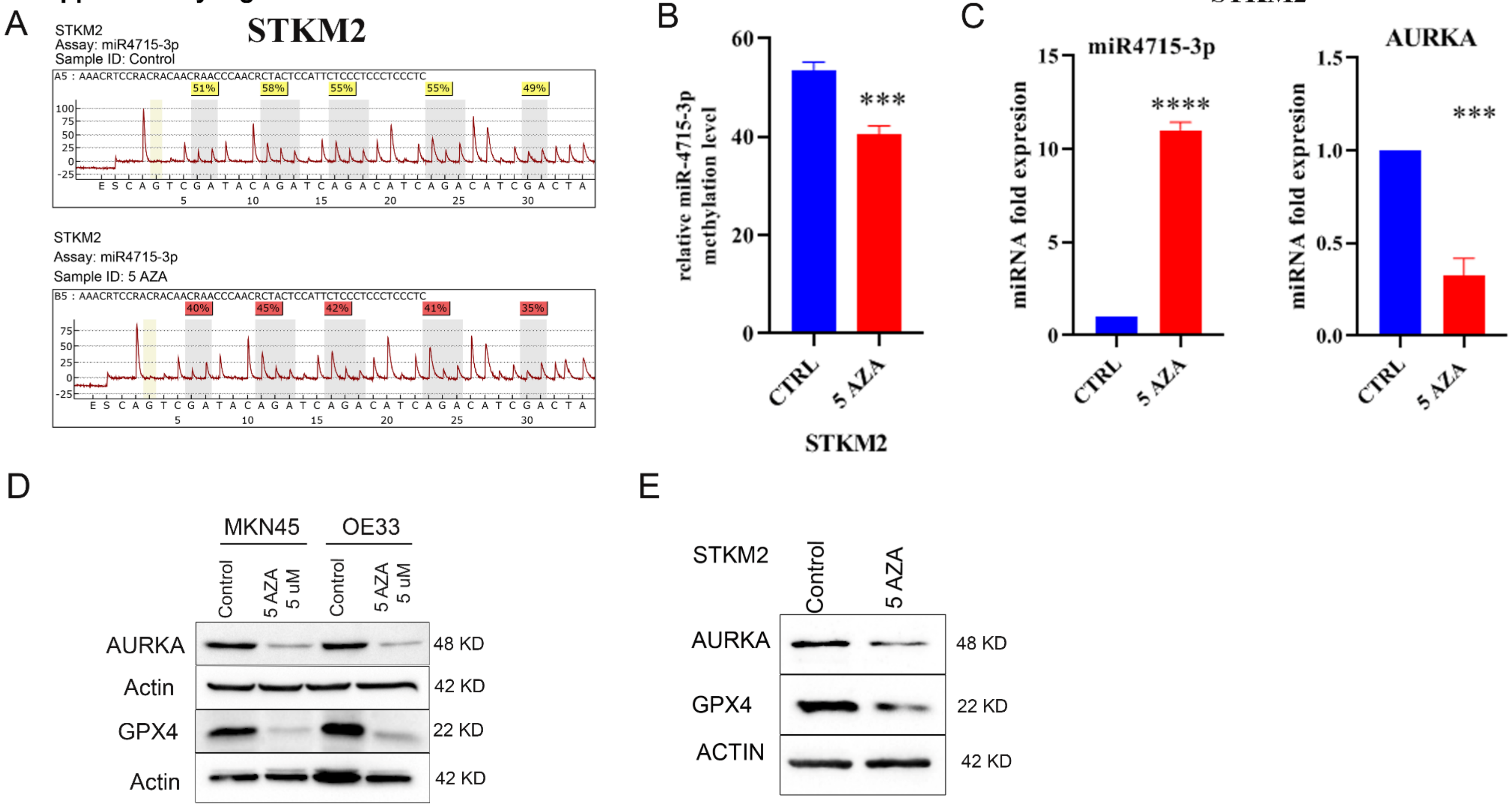

Supplementary Figure 4. (A, B) Representative pyrosequencing profiles of STKM2 cells. **(C)** AURKA mRNA and miR-4715-3p expression levels in STKM2 cells treated with 5-Aza-2'-deoxycytidine (Decitabine) 5μM for 72 h. **(D,E)** Western blot analysis of AURKA and GPX4 protein expression levels in OE33 , MKN45 and STKKM2 cells, following treatment with 5μM of 5-Aza-2'-deoxycytidine (Decitabine) for 72h.

Figure 5 A

OE33

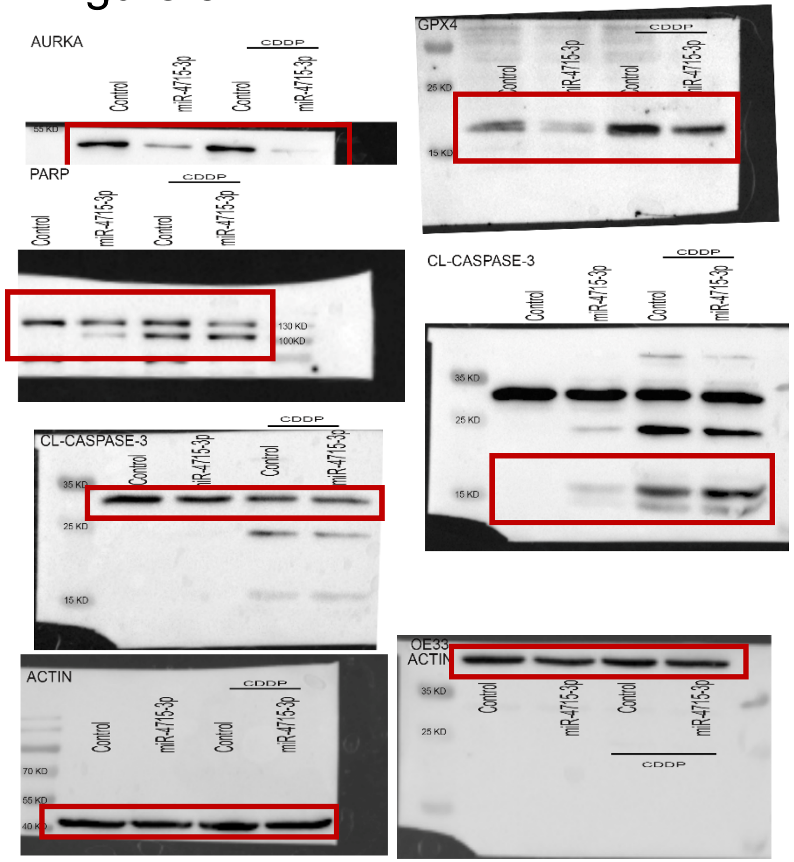

Figure 5 B

MKN45

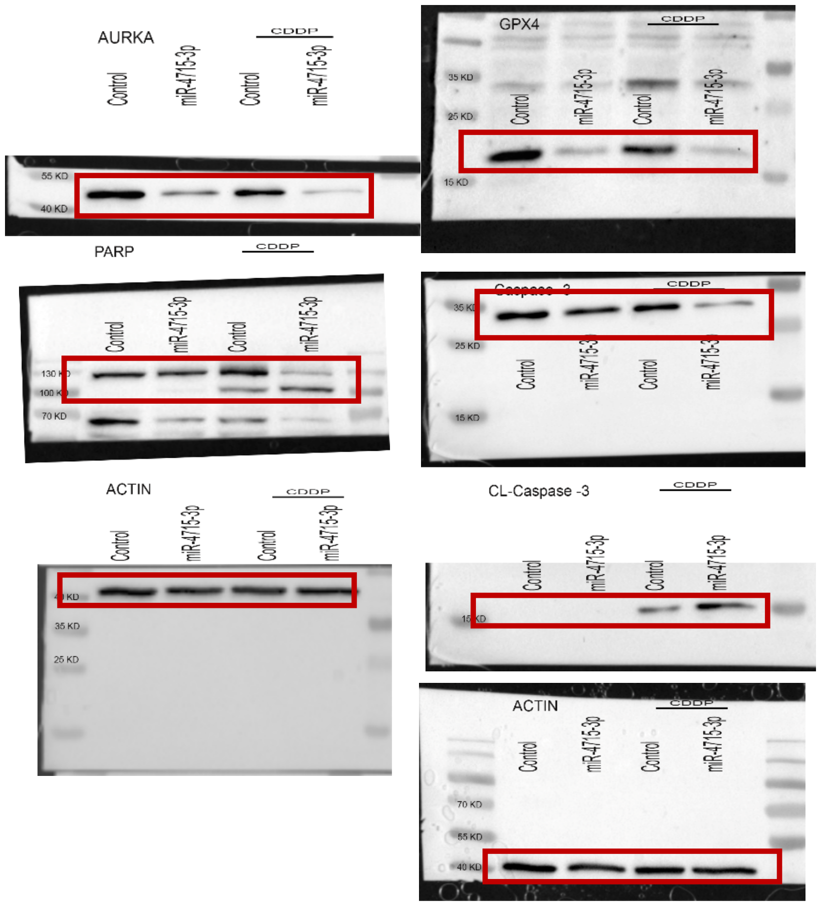

Figure 5 C

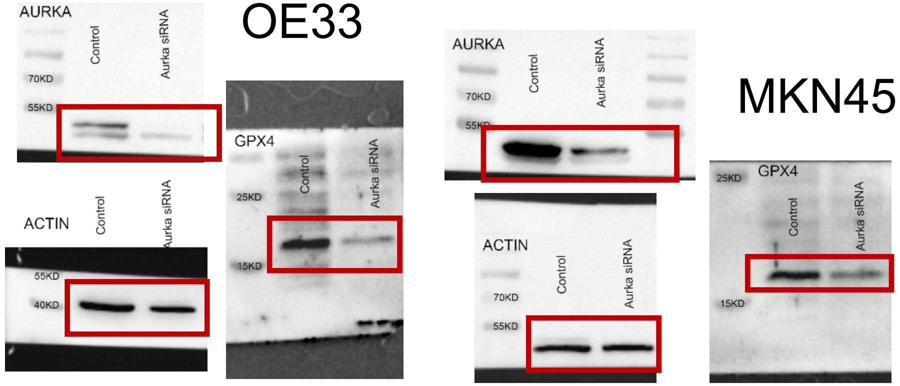

Figure 5 Raw data

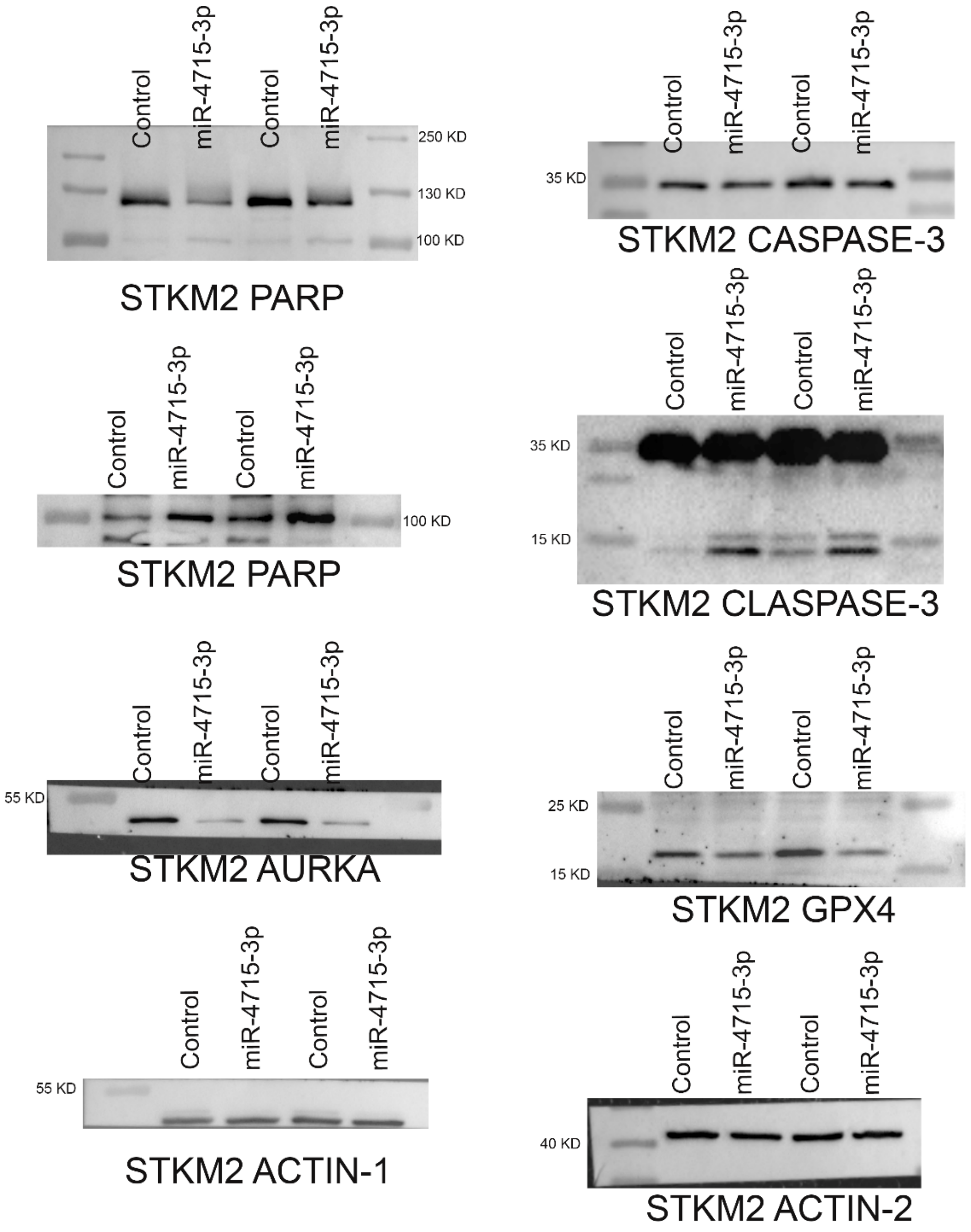

Supplementary Figure 3 Raw data

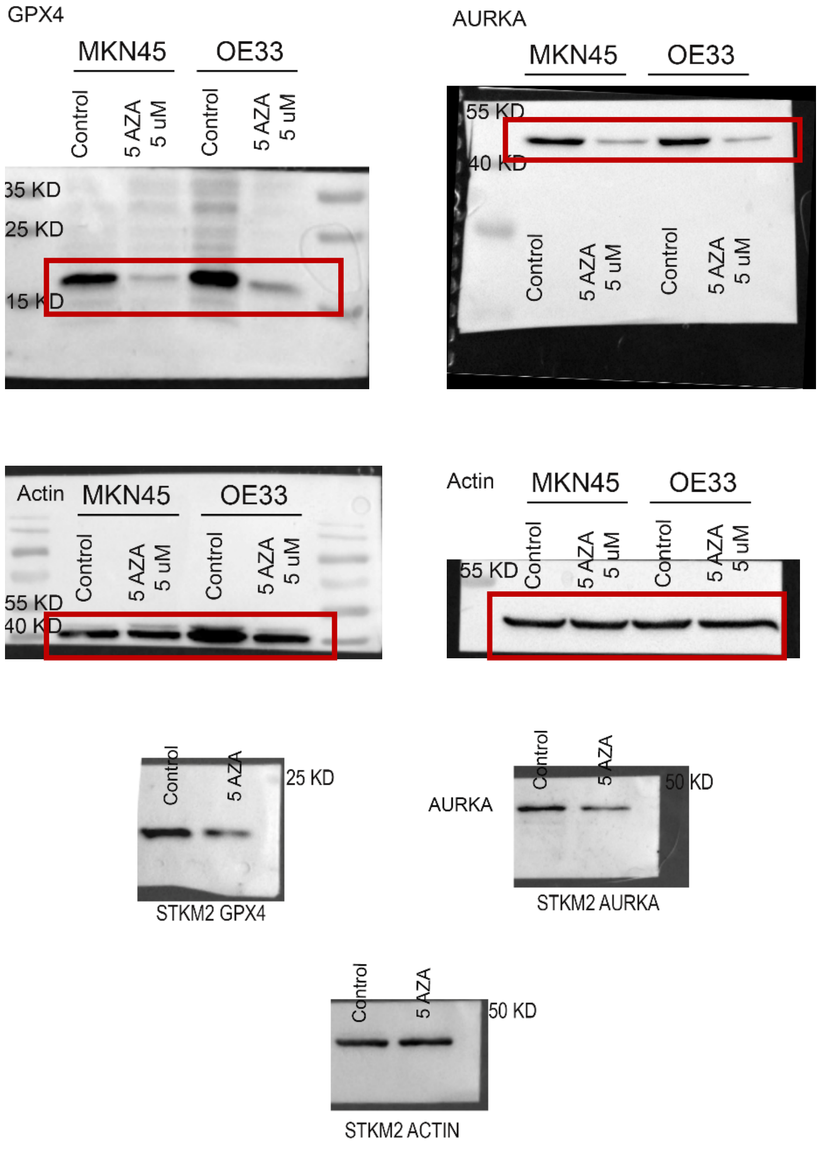

Supplementary Figure 4 Raw data
